# Neural signatures of different behavioral types in fairness norm compliance

**DOI:** 10.1038/s41598-018-28853-5

**Published:** 2018-07-12

**Authors:** Lorena R. R. Gianotti, Kyle Nash, Thomas Baumgartner, Franziska M. Dahinden, Daria Knoch

**Affiliations:** 1 Department of Social Psychology and Social Neuroscience, Institute of Psychology, University of Bern, Bern, Switzerland; 2 Department of Psychology, University of Alberta, Edmonton, Canada

## Abstract

Fairness norm compliance is critical in any society. However, norm compliant behavior is very heterogeneous. Some people are reliably fair (voluntary compliers). Some are fair to avoid sanctions (sanction-based compliers), and some are reliably unfair (non-compliers). These types play divergent roles in society. However, they remain poorly understood. Here, we combined neural measures (resting electroencephalography and event-related potentials) and economic paradigms to better understand these types. We found that voluntary compliers are characterized by higher baseline activation in the right temporo-parietal junction, suggesting better social cognition capacity compared to sanction-based compliers and non-compliers. The latter two types are differentiated by (a) baseline activation in the dorso-lateral prefrontal cortex, a brain area known to be involved in self-control processes, and (b) event-related potentials in a classic self-control task. Both results suggest that sanction-based compliers have better self-control capacity than non-compliers. These findings improve our understanding of fairness norm compliance. Broadly, our findings suggest that established training techniques that boost self-control might help non-compliers adhere to fairness norms.

## Introduction

Fairness is a central social norm in all human societies. Fairness norms are widely shared beliefs about fair behavior that promote social order, group cohesion, and cooperation. All known human societies have developed mechanisms for norm enforcement and one key mechanism is the threat of punishment^1^. Clearly, however, not everyone needs the threat of punishment to comply with fairness norms, nor do threats of punishment steer everyone uniformly towards compliance. Fairness norm compliance is characterized by heterogeneous types of behavior^2–4^. Certain people may comply freely with norms of fairness in general (*voluntary compliers*). Others may comply with fairness norms strategically to avoid punishment or social sanctions (*sanction-based compliers*). Finally, certain people may not comply with norms of fairness in general (*non-compliers*).

Despite the fact that these behavioral types may have very divergent roles and functions in society, the psychological mechanisms that underlie these different behavioral types remain largely unknown. Attempts to explain underlying psychological mechanisms usually employ self-report measures. However, self-report is susceptible to various sources of bias, including socially desirable responding, random responding, and demand characteristics^5,6^. As an alternative method, we used a neural trait approach^7^. Neural traits are objectively indexed, brain-based measures that are free from personal biases and demand characteristics. Thus, behavioral performance is left unadulterated by the act of completing trait measures and vice versa. Further, prior literature linking psychological processes to neural functioning shows that neural traits allow inferences about the psychological differences that underlie differences in decision-making. More simply, neural traits associated with certain functions can suggest both *how* and *why* people differ.

To our knowledge, prior research on the neural mechanisms of fairness norm compliance has conflated voluntary compliers with non-compliers. We suspect that this is because primary focus has been on “strategic norm compliance”, or the degree to which someone adjusts his or her behavior to avoid punishment and therefore maximize personal gain^8–13^. In these studies, fairness norm compliance is calculated in two-person distribution games as the amount of money shared with another person in a condition with the threat of punishment (*punishment threat condition*) minus the amount of money shared in a condition without a threat (*no-punishment threat condition*). Thus, the degree to which the threat of punishment induces a person to share more is strategic norm compliance because it involves compliance with a fairness norm in order to earn more money. This measure has been effective in uncovering the neural mechanisms that underlie strategic norm compliance^8–13^. However, it confounds two heterogeneous types of people, i.e., voluntary compliers and non-compliers. Specifically, a voluntary complier will demonstrate either no difference or only a small difference between the two conditions because she adheres to the norm of a fair split even in the absence of a punishment threat. A non-complier will also demonstrate either no difference or only a small difference between conditions because she adheres to pure selfishness even in the face of a punishment threat. In other words, these groups demonstrate the same score on strategic norm compliance despite very different behaviors. Unfortunately, this makes the comparison between sanction-based compliers and non-compliers in fairness norm compliance difficult to interpret because it counts voluntary compliers as non-compliers. This comparison should be considered without confounds because sanction-based compliers and non-compliers show the same behavior in the no-punishment threat condition, but only sanction-based compliers shift to compliance with the fairness norm in the punishment threat condition.

The current research sought to answer the following: Can we identify the neural signatures underlying different behavioral types in norm compliance? And, once identified, what do these signatures reveal about the psychological processes driving these three types of norm compliant behavior? Two key elements in our current study enabled us to answer these questions. First, we used a cluster analysis of transfer behavior in the two conditions (i.e., punishment threat and no-punishment threat) to separate these distinct behavioral types in fairness norm compliance. Cluster analyses can organize complex behavioral data into discrete groups based on similar behavioral patterns. An advantage of cluster analysis is that it does not require strong a priori assumptions about the data. Cluster analysis is thus ideal for the present investigation of the underlying neural signatures of distinct types in fairness norm compliant behavior. Second, we used the neural trait approach to effectively ascertain these neural signatures. This approach allows for objective measurement of meaningful dispositional differences. A neural trait can be defined as a quantifiable brain-based characteristic that is stable over time and capable of influencing preferences or behavior. Neural traits are thus much like a neural “fingerprint”, and have been used to reveal sources of individual differences in time preferences^14^, risk preferences^15^, and social preferences^9,10,16–20^.

In the present study, we collected resting electroencephalography (resting EEG) data from 184 participants. Measuring resting EEG involves recording electrical activity on the scalp when the participant is at rest to index patterns of baseline brain activation that are not related to any particular task. These patterns of baseline brain activation are ideal as neural traits because they demonstrate high stability over time^21–23^ and high specificity^22,23^ (i.e. the extent to which an EEG pattern uniquely belongs to a given person).

Compliance with the fairness norm was measured using two conditions, one in which making an unequal offer had no consequences (no-punishment threat condition), and one in making an unequal offer could be punished by the recipient (punishment threat condition). Participants played four rounds of both conditions. A participant (player A) was required to distribute 100 monetary units (MUs) (1 MU = 0.03 CHF, 100 MUs = 3 CHF) between him- or herself and the recipient (player B). In the punishment threat condition, players were informed that the recipient could either accept or punish unfair offers. Each player received an additional endowment of 25 MUs. Player B could spend all or part of the 25 MUs to reduce player A’s earnings. Every MU player B invested into punishment led to a reduction of player A’s earning by 5 MUs. In the no-punishment threat condition, player B was a passive recipient of player A’s monetary transfer. The conditions were presented in random order. Players A retained the role throughout the experiment and faced a new, randomly assigned player B in each round.

Given that this was the first study of this kind, we conducted exploratory analyses to uncover the sources of differences characterizing the different types of behavior. We expected that the neural trait approach would help explain different types of behavior in fairness norm compliance and shed light on the potential psychological processes that underlie these types.

## Results

### Emergence of three behavioral types

**Figure 1:**
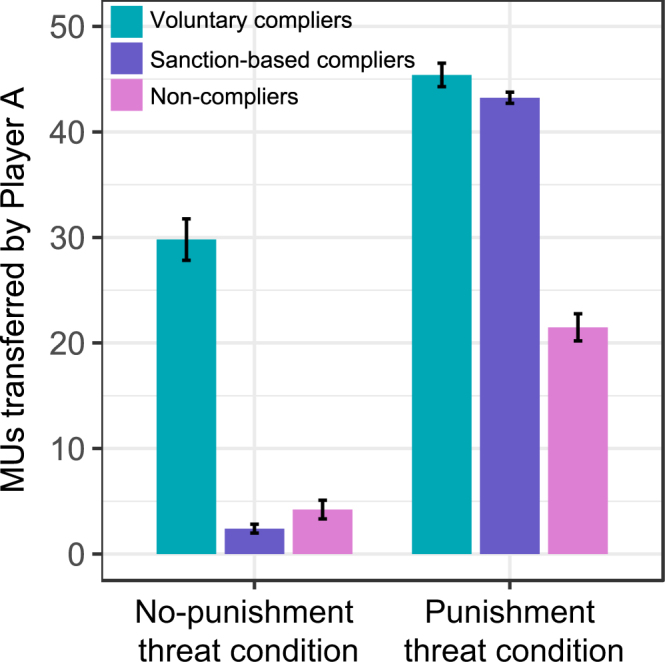
Transfer amount by condition amongst the three behavioral types. Depicted is the average amount of monetary units (±s.e.m.) transferred to the recipient in the *no-punishment threat condition* and in the *punishment threat condition*, broken down by the three behavioral types revealed by the two-step cluster analysis: voluntary compliers (turquoise; N = 25), sanction-based compliers (purple; N = 105), and non-compliers (pink; N = 54).

A two-step cluster analysis with the amount of money transferred to the recipient in the punishment threat condition and in the no-punishment threat condition entered as continuous variables revealed a solution with three separate clusters (see Fig. 1). This was also confirmed by silhouette statistic^24^ (see Supplementary Fig. 1 for the silhouette-plot of the three clusters). The first cluster included 25 individuals (13.6% of the sample, 20 female and 5 male; *voluntary compliers*) who demonstrated compliance with the fairness norm of an even-split in both conditions, i.e. they transferred 29.80 MUs (s.d. = 9.81) in the no-punishment threat condition and transferred 45.40 MUs (s.d. = 5.53) in the punishment threat condition. The second cluster included the majority of the subjects, 105 participants (57.1% of the sample, 76 female and 29 male; *sanction-based compliers*), who demonstrated compliance with the fairness norm of an even-split only under the threat of punishment, i.e., they transferred 2.40 MUs (s.d. = 4.32) in the no-punishment threat condition and transferred 43.24 MUs (s.d. = 5.41) in the punishment threat condition. Finally, the third cluster included 54 participants (29.3% of the sample, 33 female and 21 male; *non-compliers*) who demonstrated a small sanction-based increase in money transferred that yet fell well short of compliance with the fairness norm of an even-split, i.e. they transferred 4.21 MUs (s.d. = 6.48) in the no-punishment threat condition and transferred 21.48 MUs (s.d. = 9.49) in the punishment threat condition. Pearson’s Chi-square tests with Yates’ continuity correction demonstrated no significant differences in the gender-ratio between the three behavioral types (*X-squared*(2) = 3.51, *P* = 0.17).

Notably, strategic norm compliance, as evidenced by the sanction-based compliers, earned more money. Specifically, unpaired *t*-tests revealed that sanction-based compliers made significantly more money (CHF) (*M* = 22.20, s.d. = 1.28) compared to the voluntary compliers (*M* = 18.82, s.d. = 1.55, *t*(128) = 11.43, *P* < 0.001) and non-compliers (*M* = 18.58, s.d. = 1.82, *t*(157) = 14.59, *P* < 0.001). No differences were found between voluntary compliers and non-compliers (*t*(77) = 0.56, *P* = 0.58). Voluntary compliers made more money in the punishment threat condition because they were not punished by the recipients. However, non-compliers made more money in the no-punishment threat condition simply because they offered less money to the recipient.

The three types of people did not differ in age (ANOVA, *F*(2,181) = 0.198, *P* = 0.82), handedness (ANOVA, *F*(2,181) = 0.736, *P* = 0.48), positive affect (ANOVA, *F*(2,181) = 0.511, *P* = 0.60), negative affect (ANOVA, *F*(2,181) = 1.240, *P* = 0.29), or personality traits including Machiavellianism (ANOVA, *F*(2,181) = 0.602, *P* = 0.55) and Big Five personality traits (ANOVAs, all *Ps* > 0.16).

### Neural signatures of the three behavioral types

**Figure 2:**
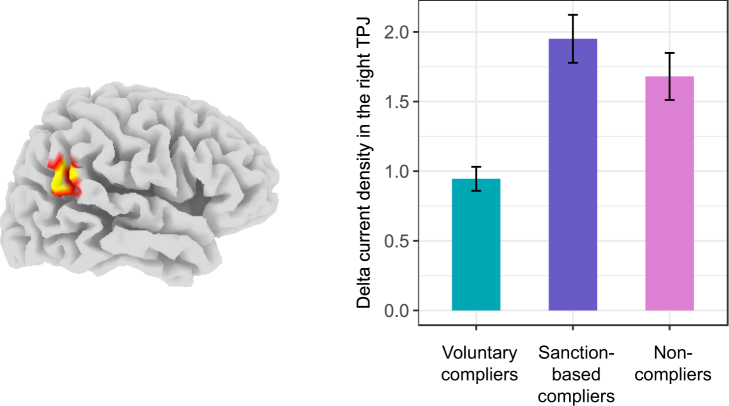
Region of the right TPJ showing differences in baseline EEG delta current density (A/m^2^) between the three behavioral types in compliance with the fairness norm. On the left side, locations of the voxels that showed significant differences (whole-brain corrected) are indicated in yellow (*P* < 0.05) or in red (0.05 < *P* < 0.10). On the right side, the bar graph (based on a 10 mm spherical ROI around the peak depicted on the left side) illustrates the baseline delta current density differences between the three behavioral types: voluntary compliers (turquoise), sanction-based compliers (purple), and non-compliers (pink). Post-hoc comparisons revealed that voluntary compliers showed significantly lower baseline delta current density in the right TPJ compared to the sanction-based compliers and non-compliers. No differences were found in the right TPJ between sanction-based compliers and non-compliers. Error bars correspond to standard errors of the means.

Our exploratory whole-brain corrected source localization analyses revealed significant differences between the three behavioral types in one specific cluster in the right temporo-parietal junction (right TPJ; MNI coordinate peak voxel: x = 65, y = −50, z = 30, Brodmann area 40; ANOVAs, all voxels: *F*(2,181) > 6.931, *P* < 0.05). As shown in Fig. 2, post-hoc comparisons revealed that voluntary compliers showed lower slow-wave delta current density in the right TPJ compared to the sanction-based compliers (*t*-test, *t*(128) = 2.82, *P* = 0.006) and non-compliers (*t*-test*, t*(77) = 2.87, *P* = 0.005). No differences were found in the right TPJ between sanction-based compliers and non-compliers (*t*-test, *t*(157) = 1.01, *P* = 0.32). Because resting slow-wave delta oscillations likely reflect decreased cortical activation^25–27^, our results indicate that voluntary compliers demonstrated higher baseline activation in the right TPJ, compared to the other two behavioral types.

**Figure 3:**
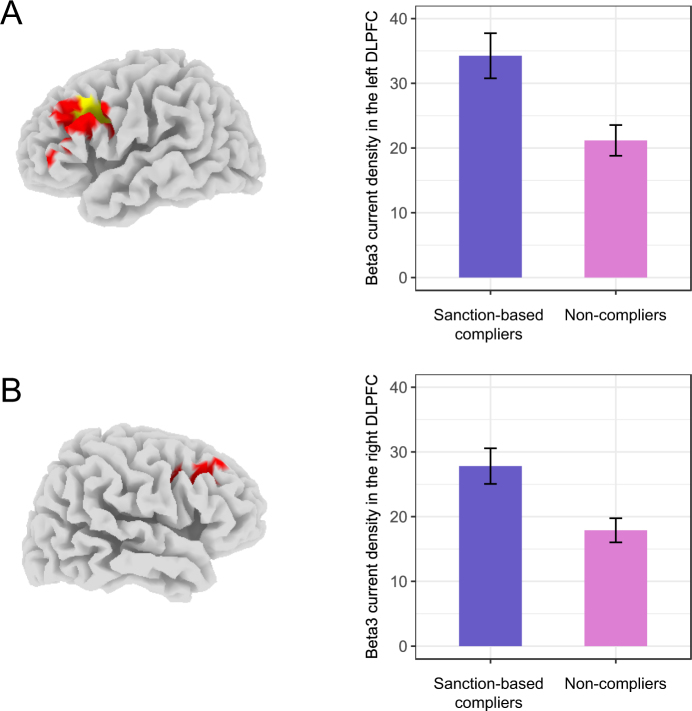
Region of the left (**A**) and right (**B**) DLPFC showing differences in baseline EEG beta3 current density (A/m^2^) between the sanction-based compliers and the non-compliers. On the left side, locations of the voxels that showed significant differences (small-volume corrected) are indicated in yellow (*P* < 0.05) or in red (0.05 < *P* < 0.10). On the right side, bar graphs (based on a 10 mm spherical ROI around the peak depicted on the left side) illustrate the baseline beta3 current density differences between the two types of people: sanction-based compliers (purple), and non-compliers (pink). Sanction-based compliers, compared to non-compliers, showed higher baseline beta3 current density in the left DLPFC (*t*-test, *t*(157) = 2.54, *P* = 0.012) as well as in the right DLPFC (*t*-test, *t*(157) = 2.44, *P* = 0.016). Error bars correspond to standard errors of the means.

How might these other two behavioral types differ, then? Recall that sanction-based compliers and non-compliers did not differ in the no-punishment threat condition—i.e., they both offered very little money. However, they showed a marked difference when behavior could be punished. Sanction-based compliers completely shifted towards fairness norm compliance in the punishment threat condition. In contrast, non-compliers shifted towards fairness norm compliance to a significantly smaller degree (*t*-test, *t*(157) = 16.63, *P* < 0.001). Thus, sanction-based compliers display more strategic behavior than non-compliers. Strategic behavior has been reliably associated with the structure and function of the dorso-lateral prefrontal cortex (DLPFC)^8–11,13^. This suggests that sanctioned-based compliers might be characterized by greater baseline activation in the DLPFC. Based on this, we applied a small-volume correction to all voxels encompassing the left and the right DLPFC (Brodmann areas 9 and 46). As shown in Fig. 3, sanction-based compliers, compared to the non-compliers, showed higher beta3 current density in the left DLPFC (MNI coordinate peak voxel: x = −35, y = 10, z = 40, Brodmann area 9; ANOVAs, all voxels: *F*(1,157) > 7.281, *P* < 0.05, small-volume corrected). In the same frequency band, a small cluster of voxels showed a similar trend in the right DLPFC (MNI coordinate peak voxel: x = 15, y = 25, z = 35, Brodmann area 9; ANOVAs, all voxels: *F*(1,157) > 5.588, *P* < 0.10, small-volume corrected). Similar results were also found in the beta2 frequency band and are reported in the Supplementary Fig. 2. Because resting fast-wave beta oscillations likely reflect increased cortical activation^28,29^, our results indicate that sanction-based compliers show more baseline activation in the right and left DLPFC, compared to non-compliers.

The DLPFC is assumed to be involved in strategic norm compliance due to its broader role in self-control^30–32^, i.e., the processes in which thoughts, emotions, or prepotent responses are inhibited to efficiently enact a more focal or appropriate goal. To properly implement strategic norm compliance, self-control should be necessary for resolving the conflict between selfish (i.e.,“keep the money”) and normative (i.e., “be fair”) motives. Sanction-based compliers, characterized by higher baseline activation in the DLPFC, may thus have increased self-control capacity to strategically adapt behavior to changing contexts.

To further probe this idea, a subsample of the sanction-based compliers (N = 95) and non-compliers (N = 51) returned for a second EEG testing session and we recorded EEG to index event-related potentials (ERPs) during response inhibition in a Go-NoGo task called the *Continuous Performance Task* (CPT). The NoGo-P300 is a well-established ERP index of response inhibition and has been reliably associated with self-control capacity^33–35^. In our CPT, participants prepared and implemented a button press to particular “target” stimuli (a Go response), as quickly as possible, and inhibited the prepared response to “non-target” stimuli (a NoGo response). As expected, we found that sanction-based compliers demonstrated larger NoGo-P300 amplitudes, compared to non-compliers (sanction-based compliers: *M* = 8.57, s.d. = 3.75; non-compliers: *M* = 6.83, s.d. = 3.21; *t*-test, *t*(144) = 2.80, *P* = 0.006). Because higher NoGo-P300 amplitudes indicate better self-control^33–35^, these results further support the idea that sanction-based compliers have an increased capacity for self-control and this increased capacity better equips them to enact strategic norm compliance.

**Figure 4:**
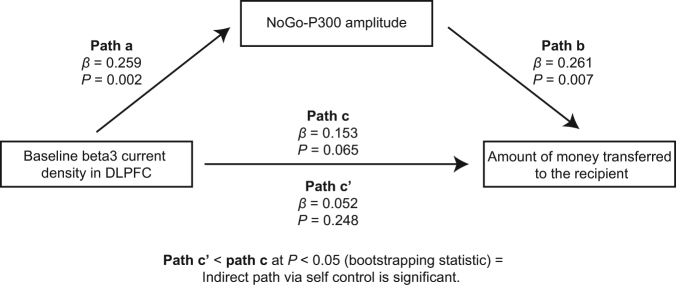
Mediation analysis. Depicted is the path diagram (including regression coefficients and *P*-values) of the mediation analysis demonstrating that the self-control capacity (indexed by the NoGo-P300 amplitude) mediates the impact of baseline activation in DLPFC (indexed by baseline beta3 current density in DLPFC) on strategic norm compliance (indexed by the amount of money transferred to the recipient in the punishment threat condition). Path a represents the effect of baseline activation in DLPFC on self-control capacity. Path b represents the impact of self-control capacity on strategic norm compliance controlling for baseline activation in DLPFC. Together, path a and path b represent the indirect (mediated) effect of baseline activation in DLPFC on strategic norm compliance through self-control capacity. Path c’ represents the direct effect of baseline activation in DLPFC on strategic norm compliance and is calculated controlling for the indirect, mediated effect. Path c represents the total (mediated and direct) effect of baseline activation in DLPFC on strategic norm compliance. Bootstrapping statistics (see Results section for details) revealed that path c’ is significantly smaller than path c (*P* < 0.05), providing evidence that self-control capacity is indeed a significant mediator.

We then tested whether NoGo-P300 amplitudes might mediate the association between baseline activation in the DLPFC and amount of money transferred to the recipient in the punishment threat condition (i.e., the condition that should involve the DLPFC and self-control). To test whether the indirect effect through a mediator is significant, bootstrapping tests for statistical significance were used^36^. We used 10000 bootstrap samples to generate bootstrap confidence intervals (95%) for the indirect effect. Results show that the indirect effect was significantly different from zero (95% CIs between 0.0086 and 0.0652, Fig. 4). This demonstrates that, consistent with the idea that sanction-based compliers are better equipped to implement strategic norm compliance, higher baseline activation in DLPFC predicts higher NoGo-P300 amplitude (i.e., increased self-control capacity), which in turn predicts more strategic behavior in social norm compliance.

## Discussion

People demonstrate remarkable variability in compliance with the fairness norm. In the current study, a cluster analysis identified three distinct types in fairness norm compliance. By using a neural trait approach, we were able to clearly differentiate these three types based on their neural signatures and shed light on the underlying psychological mechanisms. People who freely complied with the fairness norm (i.e., voluntary compliers) are characterized by higher baseline activation in the right TPJ, compared to sanction-based compliers and non-compliers. A large body of research links the right TPJ to social cognition processes such as theory of mind, empathy, and self-other distinction (for a review see^37^). Moreover, recent neuroimaging studies using fMRI show that activity in the right TPJ is positively correlated with generous behavior^38–40^ and donation behavior^41,42^. Our finding that voluntary compliers have higher baseline activation in the right TPJ is in line with this research, because voluntary compliance with the fairness norm in our study reflects a concern for others. It involved voluntarily sacrificing money to aid another person, without hope for reputational gain (the game was fully anonymous) or reciprocity (the game was a repeated one-shot setting). Our results also complement neuroanatomical research demonstrating that TPJ cortical volume impacts the propensity for altruism^19^. We ran a Spearman’s rank correlation between baseline activation in the right TPJ and the amount of money transferred to the recipient in the no-punishment threat condition. Results showed a significant negative correlation between delta current density in the right TPJ and the amount of money transferred (*rho*(183) = −0.18, *P* = 0.012; see Supplementary Fig. 3). Because resting slow-wave delta oscillations likely reflect decreased cortical activation^25–27^, this finding suggests that as baseline activation in the right TPJ increases, a participant’s propensity for altruistic behavior increases. We suggest that baseline activation in the right TPJ impacts an individual’s general propensity to behave in a fair manner, possibly due to an increased capacity for social cognition.

Not surprisingly, sanction-based compliers and non-compliers did not differ with regard to baseline activation of the right TPJ. Behaviorally, both types attempted to maximize payoffs in the no punishment threat condition by giving very little money, demonstrating an equally low concern for others. However, sanction-based compliers strategically conformed to the social norm of a fair split in the punishment threat condition to reduce the possibility of being punished, whereas non-compliers either did not make this strategic shift at all or did so to a significantly smaller degree.

Thus, despite the fact that personal gain appeared to motivate both types, only sanction-based compliers displayed a strategic shift toward norm compliance, and they were duly rewarded for this strategic shift with more money. At a neural level, sanction-based compliers were characterized by higher levels of baseline activation in the DLPFC compared to the non-compliers. Notably, the DLPFC may be involved in strategic norm compliance due to its role in self-control. Self-control is probably required to resolve the conflict between selfish (i.e., “keep the money”) and normative (i.e., “be fair”) motives to enact strategic norm compliance. Consistent with this, self-control in social behavior appears to track neuroanatomical development of the DLPFC through childhood into young adulthood^43^, and increased DLPFC activation during decision-making is associated with increased strategic decision making^8,9,11,13^. Based on this idea, we collected ERPs during response inhibition in a CPT task from both sanction-based compliers and non-compliers in a second EEG testing session. We indexed NoGo-P300 amplitude, a well-established electrophysiological measure of self-control capacity^33–35^. Mirroring the resting EEG findings, we found that sanction-based compliers demonstrated larger NoGo-P300 amplitudes, compared to non-compliers, further indicative of increased self-control capacity. Importantly, mediational analyses revealed that higher baseline activation in the DLPFC predicted higher NoGo-P300 amplitudes, which in turn predicted more strategic behavior in social norm compliance. In other words, increased DLPFC activation predicts strategic norm compliance through increased self-control capacity. Sanction-based compliers, characterized by higher baseline activation in the DLPFC, appear to have increased self-control capacity to strategically adapt behavior to changing contexts.

In sum, punishment threat is an effective method for inducing compliance with fairness norms for the majority of people (i.e. sanction-based compliers). However, punishment threat is ineffective for a sizable minority (i.e. non-compliers, roughly 30% of participants). Our results help explain why non-compliers do not react to punishment threat—i.e., they lack sufficient self-control capacity. Because of their lower self-control capacity, as evidenced by the lower baseline activation in DLPFC and by lower NoGo-P300 amplitude, they are unable to strategically adapt their behavior and adhere to fairness norms in the presence of a punishment threat. Fortunately, neural traits, though highly stable, are not immutable. We thus suggest that lasting increases in baseline activation in the DLPFC in non-compliers should improve their ability to strategically comply with the fairness norm via changes in self-control capacity. Enduring changes to specific neural traits could be made using neurofeedback, meditation, and repeated practice. These techniques can increase cortical volume or cortical baseline activation in specific brain regions^44–47^. Further, behavioral training techniques, such as working memory training^48^ or video game training^49^, can improve self-control capacity, which then helps improve important, real-world behaviors. Thus, training the sizable group of non-compliers to increase self-control capacity might also improve their ability to comply with the fairness norm. Ultimately, shifting this group of people towards improved compliance could help promote behaviors that serve the public good. These findings thus highlight the potential importance of educational programs that strengthen self-control capacity to help students become more capable to comply with social norms.

## Materials and Methods

### Participants

We collected data from 184 right-handed participants (129 female and 55 male) who were recruited from the University of Basel. All participants indicated they had no current nor previous history of neurological and psychiatric disorders or alcohol and drug abuse. Mean age was 23.1 y (s.d. = 3.1 y, range: 19–36). Participants were remunerated with a flat fee of 40 Swiss francs (CHF 1 ≈ USD 1) in addition to the money earned in the behavioral experiment. The Ethics Committee of Basel approved the study, which was conducted according to the principles expressed in the Declaration of Helsinki. All participants gave written informed consent and were informed of their right to discontinue participation at any time. All methods were performed in accordance with the relevant guidelines and regulations.

### Procedure

Behavioral and electroencephalographic (EEG) data collection took place over separate sessions. In the first session, participants completed the EEG recordings in our EEG laboratory. After obtaining written informed consent, participants completed the Positive and Negative Affect Schedule^50^ (PANAS) and a handedness inventory^51^. Participants were seated in a sound- and electrically-shielded EEG recording chamber that was dimly lit and contained an intercom connection to the experimenter. Participants were instructed about the EEG recording procedure. EEG would be recorded while they rested for 20 seconds with their eyes open, followed by 40 seconds with their eyes closed. This would then be repeated five times (such a protocol guarantees minimal fluctuations in participants’ vigilance state). Data analysis is based on the 200-seconds eyes-closed condition. In the second session, participants completed the distribution game in our behavioral laboratory with 20 interconnected computer terminals. Participants were randomly assigned to cubicles, where they could make their decisions in complete anonymity from the other participants. Every cubicle had a document with the instructions for the distribution game. The instructions were also read aloud by a student assistant. Control questions ensured participants’ understanding of the distribution game.

At the end of the behavioral session, participants filled out two personality inventories, the Machiavelli questionnaire^52^, and NEO-FFI^53^. Approximately 4–6 weeks separated the EEG session and the behavioral session. A total of 12 behavioral sessions were conducted with an average number of 16 participants per session. At the end of all behavioral sessions, participants that were identified as sanction-based compliers (N = 105) or as non-compliers (N = 54) were invited again for an EEG recording session. During this EEG session, we recorded EEG to index event-related potentials (ERPs) during response inhibition in a Continuous Performance Task (CPT).

### Distribution Game

Participants played four rounds in each of the two conditions of a distribution game. In each round, participants could split an initial endowment of 100 monetary units (MUs; 1 MU = 0.03 CHF, 100 MUs = 3 CHF) between themselves (player A) and a recipient (player B). Participants, always playing the role of player A, faced a new and randomly assigned player B in each round. The conditions were presented in random order. In one condition, which we will refer to as the *punishment threat condition*, participants were informed that the recipient could either accept or punish unfair offers. Each player received an additional endowment of 25 MUs. Player B could spend all or part of the 25 MUs to reduce player A’s earnings. Every MU player B invested into punishment led to a reduction of player A’s earnings by 5 MUs. For example, if player A decided to keep all 100 MUs for himself and player B spent all 25 MUs give player the maximum punishment, then player A’s earnings (100 MUs initial endowment plus 25 MUs additional endowment) were reduced by 125 MUs. In contrast to the punishment threat condition, in the *no-punishment threat condition*, player B was a passive recipient of player A’s distribution decisions and could not administer monetary punishment.

To generate a credible punishment threat in the punishment threat condition, the number of MUs that could be spent on punishment were calibrated in a previous behavioral pilot study (N = 23). At the end of this pilot experiment, the participants in the role of player B agreed that their decisions could be reused in other sessions of the main experiment. In the subsequent main experiment, each player A faced the decisions of those in the role of player B and hence faced decisions of real human opponents. Both players A and B were paid real money. However, player B earned money twice, once in the pilot experiment and once in the main experiment in which he was endorsed with the average transfers of player As.

### Continuous Performance Task (CPT)

This task requires the preparation and execution of responses to predefined target stimuli and the inhibition of the anticipated response to non-target stimuli. Participants were instructed to press a response button whenever the letter O (primer) is directly followed by the letter X (target; *Go-condition*). If the letter O is followed by any other letter than X (non-target), participants were instructed not to respond (*NoGo-condition*). Participants were told to give their answers as quickly and accurately as possible. The stimulus set consisted of 400 letters (12 different letters: A, B, C, D, E, F, G, H, J, L, O, and X). Of those, 80 were primer stimuli, followed by 40 target stimuli and 40 non-target stimuli. The remaining stimuli were 240 distractor letters (other letters, or X without a preceding O). Letters were presented on a computer screen in a pseudorandomized order one at a time for 200 ms with an interstimulus interval of 1650 ms. The task lasted for about 13 min.

### Electrophysiological Equipment

Continuous EEG was recorded at a sampling rate of 512 Hz (24 bit precision; bandwidth: 0.1–100 Hz) from 64 Ag–AgCl active electrodes positioned according to the 10/10 system montage^54^. During the recordings, the signals were referenced to a common-mode sense, while driven right leg served as the ground. Horizontal and vertical electro-oculographic signals were recorded with electrodes at the left and right outer canthi and left infraorbital areas. Eye movement artifacts were corrected by independent component analysis. EEG signals from channels with corrupted signals were interpolated.

### Resting EEG Data Processing

A computerized artifact rejection was applied to the EEG collected at rest (maximal allowed voltage step: 15 μV/ms; minimal allowed activity in intervals of 100-ms length: 0.5 μV; maximal allowed amplitude: ±100 μV). Data were additionally examined visually to eliminate residual artifacts (e.g. large movement-related artifacts). All available artifact-free 2048-ms EEG epochs were extracted and recomputed against the average reference. On average, there were 84.5 epochs (s.d. = 16.1) available per participant. A fast Fourier Transformation (using a square window) was applied to each epoch and channel to compute the power spectra with 0.5-Hz resolution. The spectra for each channel were averaged over all epochs for each participant. Absolute power spectra were integrated for the following 7 independent frequency bands^55,56^: Delta (1.5–6 Hz), theta (6.5–8 Hz), alpha1 (8.5–10 Hz), alpha2 (10.5–12 Hz), beta1 (12.5–18 Hz), beta2 (18.5–21 Hz), and beta3 (21.5–30 Hz).

Standardized low-resolution brain electromagnetic tomography^57^ (sLORETA) was used to estimate the intracerebral electrical sources that generated the scalp-recorded activity. sLORETA computes electrical neural activity as current density (A/m^2^) without assuming a predefined number of active sources. The sLORETA solution space consists of 6239 voxels (voxel size: 5 × 5 × 5 mm) and is restricted to cortical gray matter and hippocampi, as defined by the digitized Montreal Neurological Institute probability atlas. The sLORETA method has received considerable validation from studies combining EEG/MEG source localizations performed in conjunction with other localization methods, such as functional Magnetic Resonance Imaging^58,59^ and Positron Emission Tomography^60^. Further, the method has been validated with experimental data for which the true generators are known from invasive, implanted depth electrodes^61^. Using the automatic regularization method in the sLORETA software, we chose the transformation matrix with the signal-to-noise ratio set to 10. To reduce confounds that have no regional specificity, for each participant, sLORETA images were normalized to a total current density of one and then log-transformed before statistical analyses. Due to the larger number of female participants, we first regressed the putative sex-influence out of sLORETA images. The standardized sLORETA residuals were then used for further analyses.

### ERP Data Processing

EEG data collected during the CPT were first filtered offline with a bandpass from 0.1 to 30 Hz. After a computerized artifact rejection (only amplitudes <70 μV in all EEG channels within 200 ms before and 1000 ms after stimulus presentation were allowed), data were additionally examined visually to eliminate residual artifacts. All available artifact-free EEG epochs from correct responses were segmented, recomputed against the average reference, baseline corrected (using a −200–0 ms pre-stimulus window as baseline) and individually averaged to NoGo ERPs. All participants had at least 20 artifact-free and correct-response NoGo epochs. P300 peaks were defined as the most positive deflection within the P300 microstate (304–444 ms) at electrodes Cz. Briefly, the term *microstates* refers to short time periods of relatively stable electrical field configurations that are assumed to correspond to different steps of information processing (for further explanation of the methodology see^62^). *NoGo-P300 amplitudes* were then indexed at the respective peak latency from a cluster of 4 electrodes: Fz, FCz, Cz, and CPz for each participant.

### Statistical Analysis

The first goal of this study was to classify individual behavioral data into meaningful behavioral types to better describe the differences and similarities among individuals in fairness norm compliance. To achieve this goal we applied the two-step cluster analysis in SPSS. Two continuous variables were entered in the cluster analysis, namely the amount of money transferred to the recipient in the punishment threat condition and the amount of money transferred to the recipient in the no-punishment threat condition. The optimal number of clusters (i.e. the minimal number that best accounts for the data) is automatically determined by a two-step procedure. The first step, calculates bayesian information criterion for each number of clusters within a specified range and uses it to find the initial estimate for the number of clusters. The second step refines the initial estimate by finding the greatest change in distance between the two closet clusters in each clustering stage. Using a silhouette-plot (see Supplementary Fig. 1), we confirmed that the selected number of clusters was optimal (for details see^24^). The second and main goal of this study was to examine the neural signatures of the different behavioral types. For that purpose, we conducted whole-brain 1-way ANOVAs (separately for each frequency band) with behavioral types as the between-subject factor. The nonparametric randomization approach^63^ was used for estimating empirical probability distributions (number of randomizations used: 5000) and the corresponding critical probability thresholds (corrected for multiple comparisons).

### Data Availability

The datasets generated and/or analysed during the current study are available from the corresponding author.

## Electronic supplementary material

Supplementary Information
