# Supplementary material for: Neural signatures of different behavioral types in fairness norm compliance
Source: Sci Rep. 2018 Jul 12;8:10513. doi: 10.1038/s41598-018-28853-5 (PMC6043573; doi:10.1038/s41598-018-28853-5)
Supplement: Supplementary file 1 — Supplementary Information [file 41598_2018_28853_MOESM1_ESM.pdf]

Supplementary Information

Neural signatures of different behavioral types in fairness norm compliance

Lorena R.R. Gianotti*[1], Kyle Nash[1,2], Thomas Baumgartner[1],

Franziska M. Dahinden[1], Daria Knoch*[1]

[1]Department of Social Psychology and Social Neuroscience, Institute of Psychology,

University of Bern, Switzerland

[2]Department of Psychology, University of Alberta, Edmonton, Canada

The first two authors contributed equally to this work.

Corresponding Authors:

Daria Knoch
Dept. Social Psychology and Social Neuroscience
Institute of Psychology
University of Bern
Fabrikstrasse 8
CH-3012 Bern
daria.knoch@psy.unibe.ch
Telephone: +41 31 631 4690

Lorena R.R. Gianotti
Dept. Social Psychology and Social Neuroscience
Institute of Psychology
University of Bern
Fabrikstrasse 8
CH-3012 Bern
lorena.gianotti@psy.unibe.ch
Telephone: +41 31 631 4043

Supplementary Figure 1

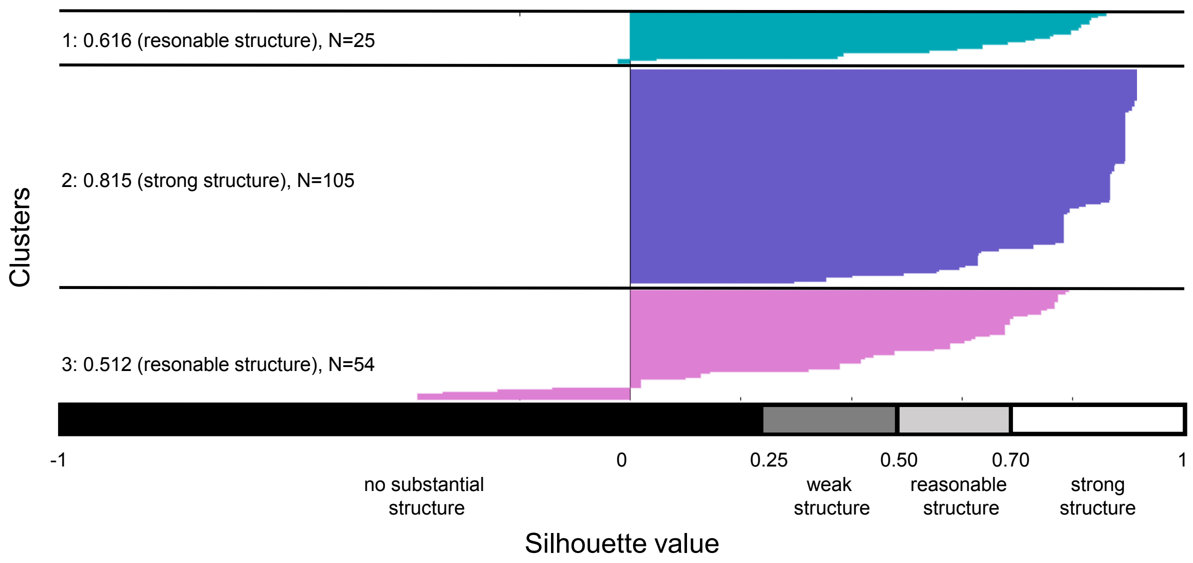

Silhouette-plot for the optimal cluster solution. Plot of silhouette values for the two-step clustering of 184 participants. On the *y*-axis all 184 participants belonging to a particular cluster are ordered by decreasing silhouette value. The *x*-axis represents the silhouette value. The silhouette value for each participant is a measure of how similar that participant is to participants in its own cluster, when compared to participants in other clusters. The silhouette value can range between −1 and 1. A high silhouette value indicates that the participant is well-matched to its own cluster, and poorly-matched to neighboring clusters. The average silhouette value and the number of participants for each cluster is given at the left. The empirical interpretation of the average silhouette value for a cluster is shown at the bottom. The colored background corresponds to the cluster assignment shown in Fig.1, that is, voluntary compliers in turquoise, sanction-based compliers in purple, and non-compliers in pink. Following the method of Rousseeuw (1987) the optimal number of clusters is selected such that the average silhouette values across all clusters is maximized.

Supplementary Figure 2

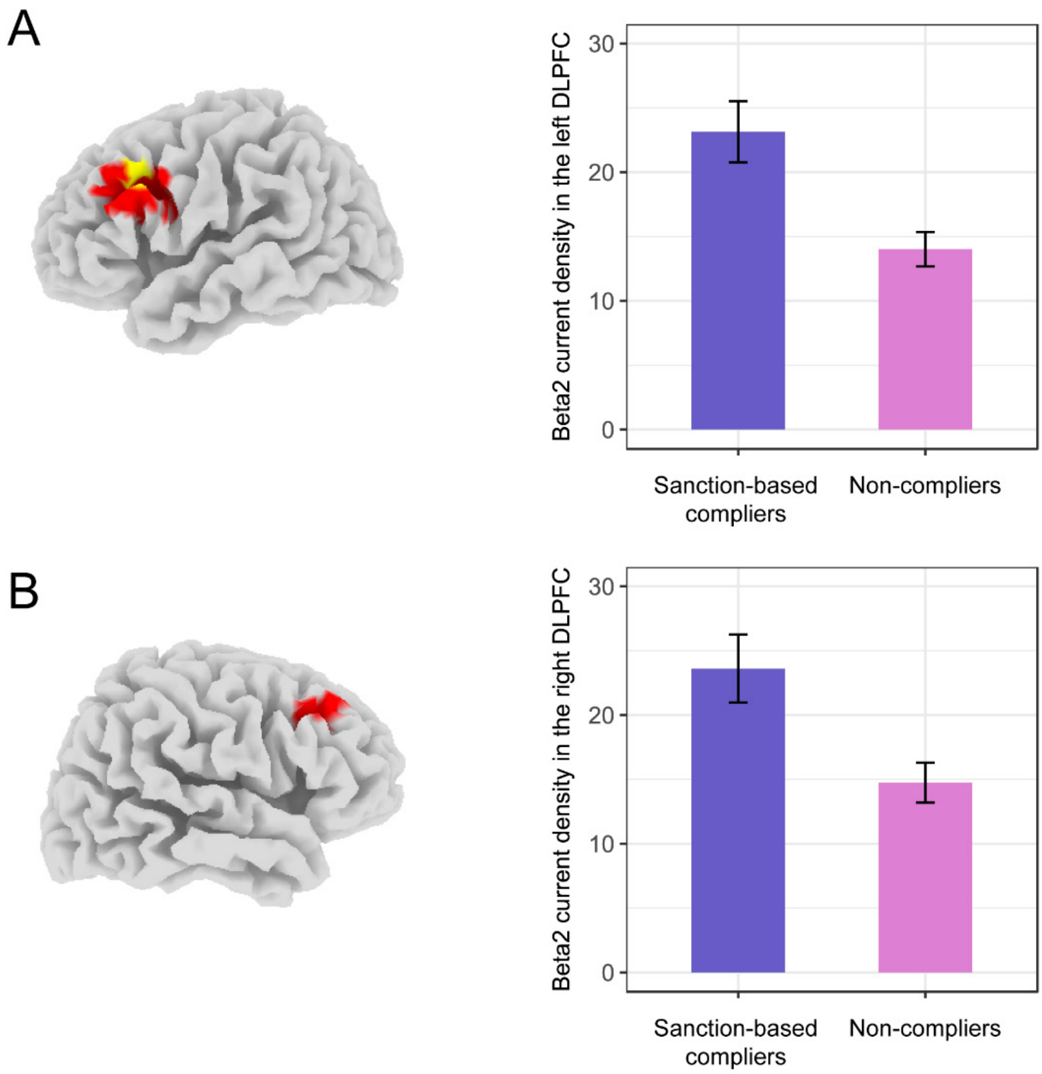

Region of the left (A) and right (B) DLPFC showing differences in baseline EEG beta2 current density (A/m^2) between the sanction-based compliers and the non-compliers. On the left side, locations of the voxels that showed significant differences (small-volume corrected) are indicated in yellow ($P < 0.05$) or in red ($0.05 < P < 0.10$). On the right side, bar graphs (based on a 10 mm spherical ROI around the peak depicted on the left side) illustrate the baseline beta2 current density differences between the two types of people: sanction-based compliers (purple), and non-compliers (pink). Sanction-based compliers, compared to non-compliers, showed higher baseline beta2 current density in the left DLPFC (*t*-test, $t(157) = 2.64$, $P = 0.009$) as well as in the right DLPFC (*t*-test, $t(157) = 2.30$, $P = 0.023$). Error bars correspond to standard errors of the means.

Supplementary Figure 3

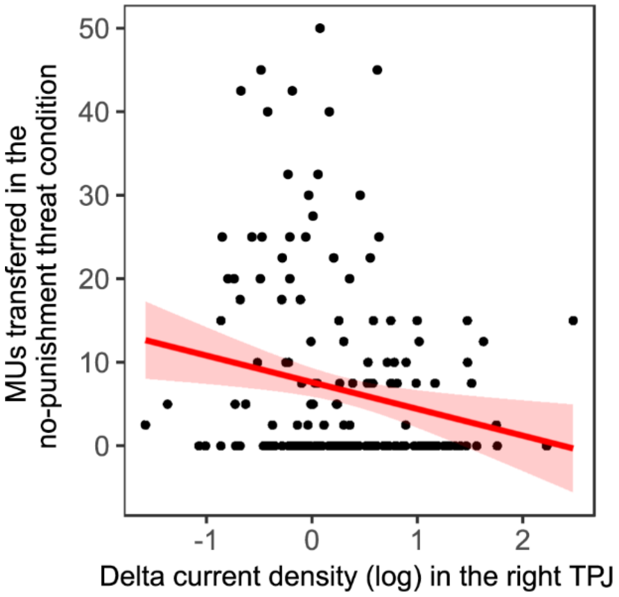

Relationship between baseline delta current density in the right TPJ and the MUs transferred by Player A in the no-punishment threat condition. The scatter plot demonstrates the negative association (*Spearman's rank* correlation, *rho*(183) = -0.18, *P* = 0.012) between baseline delta current density (log) and the amount of money transferred to the recipient in the no-punishment threat condition, including regression line (in red) and confidence intervals (95%). Because resting slow-wave delta oscillations likely reflect decreased cortical activation[1–3], this finding suggests that as baseline activation in the right TPJ increases, a participant's propensity for altruistic behavior increases.

References

1. Pizzagalli, D. A. *et al.* Functional but not structural subgenual prefrontal cortex abnormalities in melancholia. *Mol. Psychiatry* **9,** 393–405 (2004).

2. Riedner, B. A., Hulse, B. K., Murphy, M. J., Ferrarelli, F. & Tononi, G. Temporal dynamics of cortical sources underlying spontaneous and peripherally evoked slow waves. *Prog. Brain Res.* **193,** 201–218 (2011).

3. Modarres, M. H., Kuzma, N. N., Kretzmer, T., Pack, A. I. & Lim, M. M. EEG slow waves in traumatic brain injury: Convergent findings in mouse and man. *Neurobiol. Sleep Circadian Rhythm.* **2,** 59–70 (2017).
